# Supplementary material for: Comparative genomics provides new insights into the diversity, physiology, and sexuality of the only industrially exploited tremellomycete: Phaffia rhodozyma
Source: BMC Genomics. 2016 Nov 9;17:901. doi: 10.1186/s12864-016-3244-7 (PMC5103461; doi:10.1186/s12864-016-3244-7)
Supplement: Additional file 6: — List of orphan genes with links to PFAM (related to Additional file 1: Table S1). (ZIP 1428 kb) [file 12864_2016_3244_MOESM6_ESM.zip › PFAM_UNIQUE/G00541_P.html]

#                                                                            --- full sequence --- -------------- this domain -------------   hmm coord   ali coord   env coord
  
# target name        accession   tlen query name           accession   qlen   E-value  score  bias   #  of  c-Evalue  i-Evalue  score  bias  from    to  from    to  from    to  acc description of target
  
#------------------- ---------- ----- -------------------- ---------- ----- --------- ------ ----- --- --- --------- --------- ------ ----- ----- ----- ----- ----- ----- ----- ---- ---------------------
  
G00541\_P             -            789 DUF1168              PF06658.7    142       5.5    5.2  14.8   1   1     0.046        20    3.4  14.8    74   132   380   439   359   446 0.50 - Protein of unknown function (DUF1168)  
G00541\_P             -            789 MCM2\_N               PF12619.3    156       3.1    6.4  11.9   1   1     0.011       7.9    5.1  11.9    70   150   373   446   355   461 0.56 - Mini-chromosome maintenance protein 2  
G00541\_P             -            789 NARP1                PF12569.3    517       5.8    4.2   7.5   1   1     0.035       9.4    3.5   7.5   403   475   377   453   359   470 0.51 - NMDA receptor-regulated protein 1  
G00541\_P             -            789 P33MONOX             PF15302.1    294       2.8    6.1   6.0   1   1    0.0057       8.5    4.5   6.0   101   181   130   212   121   248 0.78 - P33 mono-oxygenase  
G00541\_P             -            789 Ycf1                 PF05758.7    929      0.66    6.3   5.2   1   1    0.0034      0.98    5.7   5.2   224   290   369   442   356   553 0.71 - Ycf1
